# Identification of selective inhibitors of RET and comparison with current clinical candidates through development and validation of a robust screening cascade

**DOI:** 10.12688/f1000research.8724.2

**Published:** 2016-08-23

**Authors:** Amanda J. Watson, Gemma V. Hopkins, Samantha Hitchin, Habiba Begum, Stuart Jones, Allan Jordan, Sarah Holt, H. Nikki March, Rebecca Newton, Helen Small, Alex Stowell, Ian D. Waddell, Bohdan Waszkowycz, Donald J. Ogilvie

**Affiliations:** 1 Cancer Research UK Manchester Institute, Drug Discovery Unit, University of Manchester, Manchester, M20 4BX, UK

**Keywords:** RET, KDR, Lung adenocarcinoma, Screening cascade, Selectivity

## Abstract

RET (REarranged during Transfection) is a receptor tyrosine kinase, which plays pivotal roles in regulating cell survival, differentiation, proliferation, migration and chemotaxis. Activation of RET is a mechanism of oncogenesis in medullary thyroid carcinomas where both germline and sporadic activating somatic mutations are prevalent.

At present, there are no known specific RET inhibitors in clinical development, although many potent inhibitors of RET have been opportunistically identified through selectivity profiling of compounds initially designed to target other tyrosine kinases. Vandetanib and cabozantinib, both multi-kinase inhibitors with RET activity, are approved for use in medullary thyroid carcinoma, but additional pharmacological activities, most notably inhibition of vascular endothelial growth factor - VEGFR2 (KDR), lead to dose-limiting toxicity. The recent identification of RET fusions present in ~1% of lung adenocarcinoma patients has renewed interest in the identification and development of more selective RET inhibitors lacking the toxicities associated with the current treatments.

In an earlier publication [Newton *et al*, 2016; 1] we reported the discovery of a series of 2-substituted phenol quinazolines as potent and selective RET kinase inhibitors. Here we describe the development of the robust screening cascade which allowed the identification and advancement of this chemical series.  Furthermore we have profiled a panel of RET-active clinical compounds both to validate the cascade and to confirm that none display a RET-selective target profile.

## Introduction

RET is a receptor tyrosine kinase (TK) expressed primarily on derived neural crest and urogenital cells during embryonic development. It is required for maturation of several cell lineages of the peripheral nervous system, kidney morphogenesis and spermatogenesis ^2^. The glial derived neurotrophic factor (GDNF) family of ligands bind RET in association with one of four glycosyl phosphatidylinositol (GPI) anchored GDNF family α-receptors (GFRα), triggering RET dimerization, followed by auto-phosphorylation of specific tyrosine residues within the C-terminal chain and trans-phosphorylation of intracellular signalling cascades. These downstream signalling networks play a key role in regulating cell survival, differentiation, proliferation, migration and chemotaxis ^3^.

Activating mutations in *RET* ( *e.g.* C634W and M918T) have been identified in familial and sporadic forms of medullary thyroid carcinomas (MTC ^4, 5^) and are associated with aggressive disease progression ^6^. More recently, several groups independently identified RET rearrangements in 1–2% of lung adenocarcinoma (LAD) cases ^7– 10^. The *RET* fusion genes discovered in these studies include CCDC6-RET (already known as RET/PTC1 in papillary thyroid carcinoma) as well as a novel fusion with KIF5B (kinesin family member 5B), encoding a coiled coil domain, generated by pericentric inversion in chromosome 10. The coiled-coil domains present in the fusion partner promote overexpression and ligand-independent dimerization leading to constitutive activation of RET. These studies also demonstrated that the resulting fusion proteins are oncogenic, and that their inhibition has therapeutic potential. Importantly, the RET fusions are mutually exclusive with other known drivers in LAD (e.g. KRAS, epidermal growth factor receptor (EGFR), EML4-anaplastic lymphoma kinase (ALK)), further supporting a role for RET as a unique driver of malignancy in these tumors. RET-positive patients represent a well-defined population with specific features: all are adenocarcinomas, and patients tend to be non-smokers and to be younger than the median age for lung cancer patients ^11^.

At present, there are no known specific RET inhibitors in clinical development, although many potent inhibitors of RET have been opportunistically identified through selectivity profiling of compounds initially designed to target other TKs. The small molecule inhibitors vandetanib and cabozantinib are perhaps the best examples of such compounds. Although both have been approved for the treatment of advanced metastatic MTC ^12, 13^, RET inhibition is a secondary pharmacology of these drugs, which were initially developed as inhibitors of other TKs. Both agents target KDR, whilst vandetanib has additional activity versus EGFR and cabozantinib versus MET. These compounds are now under investigation for the treatment of RET fusion positive LAD. A preliminary report of a phase II trial ^14^ of cabozantinib confirmed partial responses in two of three RET-positive patients ^11^; the third patient presented with prolonged stable disease. The activity of vandetanib in RET fusion positive patients has been demonstrated in two case reports ^15^. However significant toxicity ( *e.g.* rash, diarrhoea, hypertension) resulting from inhibition of other kinases, particularly KDR, has led to dose reductions in clinical trials (11–13) and is likely to compromise the use of both these agents in clinical settings ^16^. Thus, there is a clear need for selective RET inhibitors which do not display the toxicities associated with the current treatments and enable more potent and sustained inhibition of RET signalling. These agents may offer greater clinical benefit for patients with RET mutant cancers and widen the scope for the clinical use of RET inhibitors ^17^.

The role of RET in this subset of LAD has heightened interest in re-purposing a number of other clinically approved inhibitors, shown to have RET activity in pre-clinical studies. Sunitinib, sorafenib, ponatinib and lenvatinib, all multi-kinase TK inhibitors with some RET activity, are currently under investigation in numerous phase II clinical trials ^14^ for treatment of RET fusion positive LAD ^18^. Sunitinib, already approved for the treatment of imatinib-resistant gastrointestinal stromal tumors (GIST), advanced renal carcinoma and advanced pancreatic neuroendocrine tumors, is the subject of a phase II study in certain types of LAD tumors, including those harbouring a RET fusion. Sorafenib, also approved for several indications including kidney and liver cancer, has demonstrated preclinical activity in RET models but has yet to be tested in patients selected based on RET fusion status. Some efficacy in advanced MTC has been reported for lenvatinib ^19^, however tumor response did not correlate with RET mutation status and the observed toxicity profile was consistent with KDR inhibition. A phase II study of lenvatinib in RET fusion positive LAD is ongoing ^14^. Ponatinib is also a multi-targeted, broad-spectrum tyrosine kinase inhibitor ^20^, approved in late 2012 for patients with resistant or intolerant chronic myeloid leukemia and Philadelphia chromosome-positive acute lymphoblastic leukemia. Ponatinib was withdrawn shortly afterwards due to serious safety concerns but was later returned to the market with additional warnings in the product information. An investigational phase II clinical trial of ponatinib in LAD patients selected based on RET mutation status is currently ongoing ^14^. Alectinib is a highly selective ALK inhibitor (median inhibitory concentration of 1.9 nM for ALK activity), recently approved by the FDA for the treatment of patients with ALK positive LAD who progressed on crizotinib. Preclinical data demonstrating activity of alectinib in RET mouse models ^21^ has led to its investigation as a treatment for RET fusion positive LAD as part of the DARWIN II trial ^14^. The compounds described above are all classic TK inhibitors but in 2010, Alfano *et al.* ^22^ proposed an alternative approach for targeting RET in LAD involving inhibition of HSP90 (heat shock protein 90kDa). HSP90 is a molecular chaperone that plays a central role in regulating the correct folding, stability and function of numerous proteins ^23^. Inhibition of HSP90 activity results in aggregation or proteasomal degradation of these proteins. RET, along with other driver kinases such as EGFR and ALK is a HSP90 client protein and as such requires HSP90 for protein stability and function. Thus, targeting the chaperone function of HSP90 offers an alternative to direct kinase inhibition for therapeutic intervention in RET driven cancer. Clinical evaluation of this approach is currently being assessed as part of a larger study in stage IV LAD patients with driver molecular alterations other than EGFR mutations ^14^. **

In an earlier publication ^1^ we reported the discovery of 2-substituted phenol quinazolines as potent RET kinase inhibitors with improved KDR selectivity; here we describe development of the robust screening cascade which allowed us to achieve that goal and additionally profile existing clinical compounds with RET activity in order to assess them against our compound target profile.

## Materials and methods

### Material

Clinical compounds were purchased from SelleckChem. Paterson Drug Discovery (PDD) compounds were synthesised in-house by methods described in an earlier publication ^1^. All compounds were dissolved at 20mM in dimethyl sulfoxide (DMSO, Sigma) and stored at -20°C or in a desiccator at room temperature.

### Biochemical kinase assays

RET and KDR kinase activities were measured according to methods previously described using the HTRF kinEASE kit (CisBio) ^1^. For measurement of mutant RET (M918T; Carna Bioscience), the following modifications were made: 50pM RET (M918T), 9µM ATP and 1µM substrate. For the slow binding studies, the standard kinase assay procedure was used but with varying time of compound pre-incubation with RET prior to the addition of ATP. Reversibility of binding was determined by measuring the recovery of enzymatic activity after a rapid and large dilution of the enzyme-inhibitor complex. RET enzyme was incubated at 100 fold the concentration normally required for the standard screening assay, with a concentration of inhibitor equivalent to 10 fold the IC _50_. After 15 minutes of incubation, this mixture was diluted 100 fold in the reaction buffer containing the enzyme substrates to start the reaction. This diluted the enzyme to the standard assay concentration and the compound from 10 fold to 0.1 fold of the IC _50_ concentration. Theoretically, following this 100-fold dilution of the pre-incubation mixture, 91% enzyme activity will be recovered for a fully reversible inhibitor. However, taking into account assay variability, compounds showing >75% enzymatic activity recovery compared to the no inhibitor positive control were classed as fully reversible, those with <75% but where recovery of some activity was clearly detected were classed as slowly reversible *(i.e.* would eventually reach >75% recovery of activity). To test whether compounds were ATP competitive, assays were performed under standard conditions with 9µM ATP (Km), and then repeated at 450µM ATP (50× Km). By increasing the amount of ATP in the reaction by 50 fold, the IC _50_ value should increase for competitive compounds, theoretically by a ratio of 25. For non-competitive compounds the ratio should be 0.5.

### Cell culture

MZ-CRC-1 (gift from Alexander Knuth, University of Zurich) LC-2/ad (RIKEN) and HEK293 (ATCC) cells were cultured in advanced DMEM/F12 media (Invitrogen), supplemented with 5% fetal bovine serum (FBS, Invitrogen) and 2mM Glutamax (Invitrogen) and incubated at 37°C in 5% CO _2_/air. The BaF3 cell lines, expressing KIF5B-RET (gift from Pasi Janne ^10^) and KDR (Advanced Cellular Dynamics, San Diego) were maintained in RPMI-1640 (Invitrogen) media containing 10% Hyclone FBS (Scientific Laboratory Supplies) with the addition of 1µg/mL puromycin (Sigma) for the KDR cell line. Non-modified BaF3 cells (WT; DSMZ, Germany) were maintained in RPMI-1640 media (Invitrogen) containing 10% FBS (Invitrogen) and supplemented with 10 ng/mL recombinant mouse IL-3 (R&D systems).

### Cell POM (proof of mechanism) assay

The RET proof of mechanism assay (POM) assay measures the compound effect on the target. Active forms of RET and KDR are phosphorylated and thus compound inhibitory activity may be measured directly by quantifying levels of the phosphoproteins, pRET and pKDR, remaining after cell treatment. MZ-CRC-1 cells harbour the M918T mutation and thus express constitutively high levels of pRET and although pKDR is barely detectable it is possible to increase expression with ligand stimulation thus allowing measurement of RET and KDR inactivation within the same cell lysate. MZ-CRC-1 cells were seeded into 96-well plates at 100,000 cells per well in 100µL culture medium. After 24 hours, medium was replaced with 100µl of serum-free medium and cells were incubated overnight. Compounds were dispensed into the appropriate wells using an acoustic liquid handling platform (LABCYTE). Cells were incubated with compound for 2h, followed by vascular endothelial growth factor (human VEGF 165, R&D Systems) ligand stimulation (50ng/ml for 5 minutes at 37°C). VEGF treatment does not affect levels of pRET (data not shown). Cells were washed with 100µL ice-cold PBS and 30µL lysis buffer (Cell Signalling Technology) added. Plates were incubated at 4°C for 1 hour, resulting lysates were transferred to another 96-well plate and protein concentration determined using the Millipore Direct Detect infrared spectrometer. Levels of pRET and pKDR in the cell lysates were determined, according to the manufacturer’s instructions, using the pRET (panTyr) and pVEGFR-2 (Tyr1175) PathScan sandwich ELISA kits (Cell Signalling Technology). Compound IC _50_s were based on levels of phosphoprotein normalised to control values. Selectivity (versus KDR) was calculated by dividing KDR IC _50_ value by RET IC _50_ value.

 Compound effects on pRET levels in LC-2/ad cells were measured as described above except that 30,000 cells per well were plated and the ligand stimulation step was omitted; KDR protein is not detectable in this cell line and therefore it is not possible to determine compound effects on levels of pKDR.

### Cell POP (proof of principle) assay

The RET proof of principle (POP) assay measures compound effects on cell proliferation in a disease relevant model. For routine screening we compared anti-proliferative effects of the compounds in the disease model, MZ-CRC-1 (RET (M918T)) versus a control, non-RET expressing cell line, HEK293. This allows us to demonstrate translation of mechanistic effects measured in the POM assay into RET-specific phenotypic effects in cells. MZ-CRC-1 and HEK293 (control, no RET expression) cells were seeded into 384 plates at 4000 and 1000 cells per well respectively in 30µL culture medium and confluence monitored at 4 hourly intervals using the IncuCyte ZOOM live cell imaging platform (Essen). After 48 hours, compounds were dispensed as described above and cells incubated until confluence of control cells reached 80–90%. Compound IC _50_s were calculated based on cell confluency at this time point normalised to control values. Non-specific toxicity margin was calculated by dividing IC _50_ value obtained for the HEK293 control cells by that for the MZ-CRC-1 cells.

The POP assay has also been used to measure compound effects in other disease relevant cell lines, for example, LC-2/ad, an LAD cell line harbouring the CCDC6-RET fusion. LC-2/ad cells were seeded into 96 well plates at 4000 cells per well in 100µL serum free culture medium and incubated at 37°C in 5% CO _2_. After 48 hours compounds were diluted to 2x final concentration and added to appropriate wells in 100µL serum-free culture medium. Once control cells had reached 80–90% confluence, protein content was measured using the sulforhodamine B (SRB) assay ^24^. Compound IC _50_s were based on protein content (proportional to cell number) normalised to control values.

### BaF3 POM assay

This assay was performed as described previously ^1^. Selectivity values were calculated as described above.

### Western blotting analysis

Following compound treatment (1µM, 24 hours), MZ-CRC-1 cell lysates (50 μg) were subjected to polyacrylamide gel electrophoresis (PAGE) and semi-dry transfer to nitrocellulose membrane using the Bio-Rad Trans-Blot Turbo system and Trans-Blot Turbo transfer packs. Membranes were blocked overnight at 4°C in phosphate-buffered saline containing 0.1% Tween (PBST; Sigma) and 5% non-fat dried milk powder (marvel). Primary and secondary antibody incubations were performed at room temperature in PBST/0.5% marvel with 3× PBST washes post incubation. pRET (Santa Cruz #SC-20252-R) and total RET (Santa Cruz #sc-167) antibodies were used at 1:500 dilutions; GAPDH (Cell Signaling Technology #2118L) and secondary goat anti rabbit IgG HRP-linked (Cell Signaling Technology #7074) antibodies were used at 1:1000. Proteins were visualized by chemiluminescent detection of peroxidase activity using SuperSignal reagent (Pierce), and images were captured using the Syngene imager and GeneSys software.

## Results and discussion

**Figure 1.**
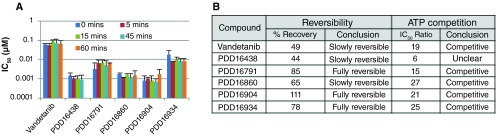
Mechanism of inhibition data. ( **A**) Effect of increasing pre-incubation time (5–60 mins) on compound IC _50_ value. ( **B**) Table showing results of reversibility and ATP competition studies.

Our aim was to develop and validate a robust screening cascade to support the identification and development of potent and selective RET inhibitors using vandetanib as the starting point ^1^ and to assess these attributes in clinical compounds reported to have RET activity. All compounds were initially assayed biochemically for activity versus RET and KDR enzyme. Once potency and selectivity had been confirmed, and structure activity relationships (SAR) demonstrated, for a number of compounds within the anilinoquinazoline series ^1^, we performed further biochemical studies to investigate the mechanism of RET inhibition using selected compounds including our starting point, vandetanib ( Figure 1). Some inhibitors bind to, or dissociate from the target enzyme slowly, leading to time dependent inhibition ^25^. Failure to identify this can lead to an underestimation of biochemical potency and misleading SAR. To investigate this, compounds were assayed under standard conditions but pre-incubated with RET for between 0 and 60 minutes prior to addition of ATP. Pre-incubation did not significantly affect IC _50_ values indicating that the anilinoquinazolines are not slow binders and that a 15 minutes pre-incubation, as used in our standard assay, is sufficient to allow the reaction to reach equilibrium ( Figure 1A). Although diverse in primary amino acid sequence, the human kinases share a great degree of similarity in their 3D structures, especially in their catalytically active kinase domain where the ATP-binding pocket is located. Kinase inhibitors can be grouped into two classes, based on binding mode: irreversible and reversible. The former tend to bind covalently with a reactive nucleophilic cysteine residue proximal to the ATP-binding site, resulting in the blockage of the ATP site and irreversible inhibition. Reversible inhibitors can be further classified into four main types, competitive, non-competitive, uncompetitive and mixed inhibition ^26^. Our data ( Figure 1B) indicates that the anilinoquinazolines under investigation here fall into the reversible, ATP competitive category which is not surprising since it has been demonstrated previously that vandetanib, a related compound and our starting point, exhibits this mode of binding ^27^.

Compounds meeting defined criteria for potency and selectivity in the biochemical assay were then assessed in a cellular POM assay. Initially we developed an ELISA-based POM assay, measuring changes in levels of (active) phosphorylated RET (M918T) and KDR, within the same MTC cell line (MZ-CRC-1).

**Figure 2.**
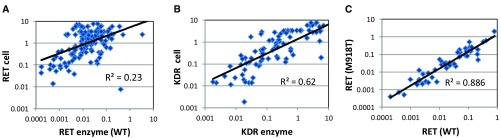
Correlation of IC _50_ data: **A**) RET enzyme vs RET cellular POM **B**) KDR enzyme vs KDR cellular POM **C**) RET enzyme WT vs RET (M918T). All data points represent the geometric mean of at least two independent measurements.

**Supplementary Figure 1.**
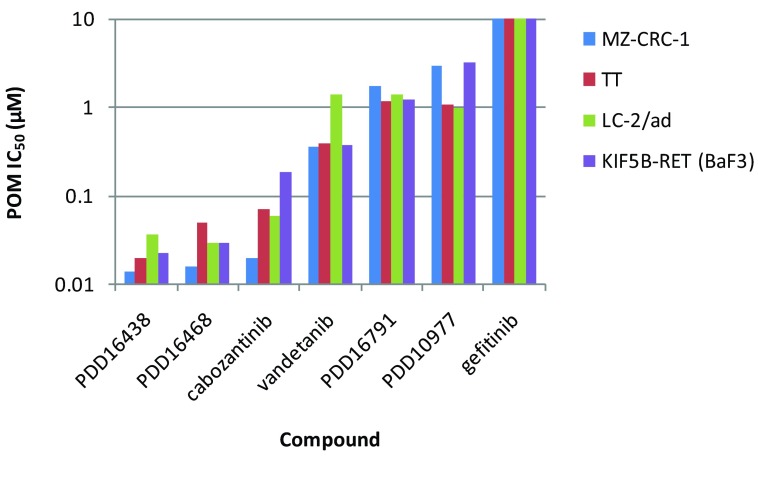
Comparison of POM IC _50_ data across RET cell models. All data points represent the geometric mean of at least two independent measurements. IC _50_ data for MZ-CRC-1, LC-2/ad, TT and KIF5B-RET (BaF3) cells.

However, it soon became apparent that compared to KDR there was a poor correlation between RET biochemical and cell-based IC _50_ measurements ( Figures 2A & B). In addition, the drop off in potency from the biochemical to cellular assay was generally much greater for RET than KDR, in effect compressing the selectivity margins, often from >100-fold in the biochemical assay to parity (or worse) in the cellular assay. Compound permeability was not believed to be the cause of the disparity as the magnitude of the drop off was not the same for both RET and KDR, despite measurement of both proteins in the same cell lysate. A previous report ^28^ had indicated that a single mutation within the kinase domain of RET could alter compound inhibitory activity. In order to address the possibility that some of the discrepancy could be due to our measurement of WT protein activity biochemically but mutant RET (M918T) protein in the MZ-CRC-1 cells, we compared the biochemical activity of selected compounds versus both RET (WT) and RET (M918T) protein. The compound IC _50_ data for the two proteins correlate well ( Figure 2C) and therefore indicate that the mutation does not significantly affect biochemical activity for this class of compound. In order to follow this up in the cellular context we extended our repertoire of assays to enable robust measurement of RET activity in TT ^29^, LC-2/ad ^30, 31^ and mouse BaF3 ^10^ cells harbouring a C634W mutation, a CCDC6-RET fusion and a KIF5B-RET fusion respectively. We assessed relative RET potencies for a number of tool compounds (including vandetanib and cabozantinib) across these cell lines and found that activities correlated well ( Supplementary Figure 1), further supporting the notion that this class of compounds are equally active versus mutant and WT forms of the protein.

**Figure 3.**
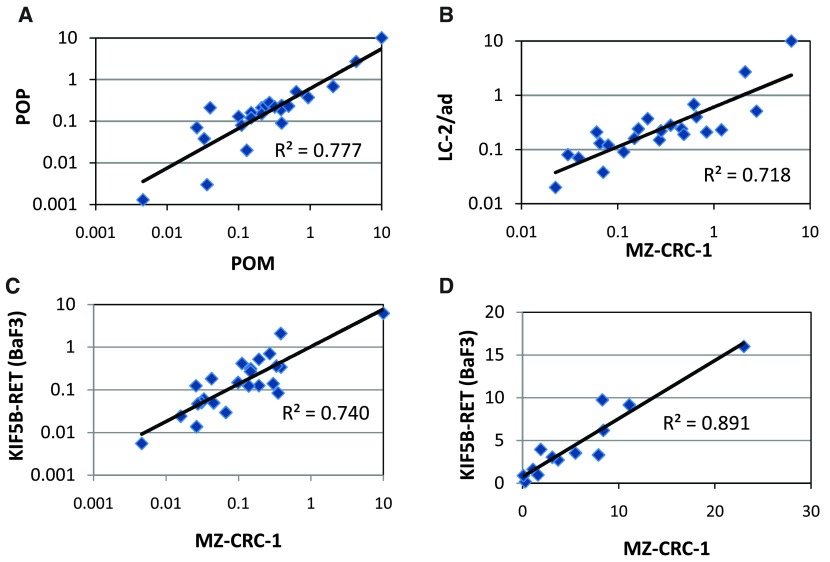
Correlation of cellular data: **A**) POM vs POP IC _50_ data in MZ-CRC-1 **B**) POP vs POP IC _50_ data in MZ-CRC-1 and LC-2/ad **C**) POM vs POM IC _50_ data in MZ-CRC-1 and KIF5B-RET (BaF3) **D**) POM vs POM selectivity data in MZ-CRC-1 and KIF5B-RET (BaF3). All data points represent the geometric mean of at least two independent measurements.

**Supplementary Figure 2.**
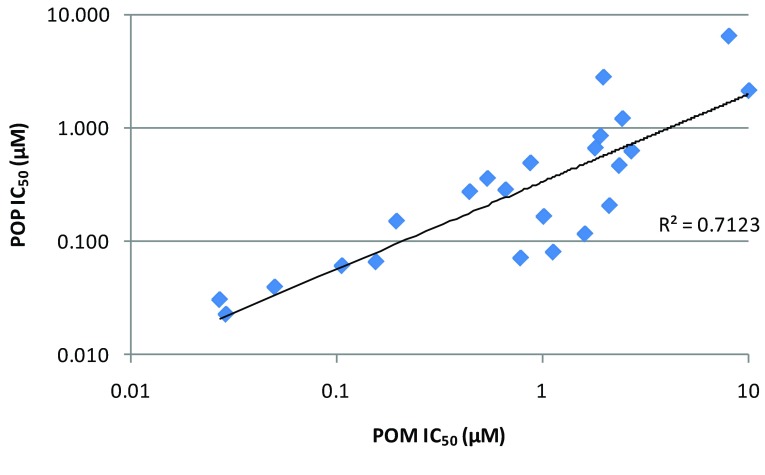
Correlation of POM and POP IC _50_ data generated in the LC-2/ad cell line. All data points represent the geometric mean of at least two independent measurements.

In order to demonstrate POP in a disease relevant cell line, we developed and validated a proliferation endpoint assay in the MTC line MZ-CRC-1; non-specific toxicity was evaluated by measuring the same endpoint in HEK293, a human embryonic kidney line which does not express RET. Compounds exhibiting RET potency (<500nM) and selectivity (>10x) in the POM assay were selected for POP assay screening. Our data show that there is a good correlation between the POM and POP IC _50_ values measured in the MZ-CRC-1 ( Figure 3A) cells and other RET models (e.g. LC-2/ad, Supplementary Figure 2) indicating that the RET inhibitory activity that we observe in our POM assay translates into anti-proliferative effects in our MTC and LAD disease models. In addition, we have assayed a selection of representative compounds for anti-proliferative effects in LC-2/ad and as for the POM assay, data correlate well with that obtained in MZ-CRC-1 ( Figure 3B). Therefore, we are confident that activities observed in our routine MZ-CRC-1 POM and POP screens are predictive for the other cell line models.

Although at this stage we were confident that we could drive our internal chemistry effort based on the cellular data, it was clear that performance in the biochemical assay was not always a good predictor of RET cellular activity and therefore should not be used as a pre-screen for this chemical series. To address this issue this we developed and validated a higher throughput POM assay allowing parallel assessment of all compounds in both cellular and biochemical assays. This BaF3 proliferation assay platform, employing cell lines dependent upon RET or KDR for survival alongside WT IL-3 dependent control cells, is robust with 4x higher throughput than the original MZ-CRC-1 POM ELISA. More importantly the proliferation IC _50_ for both RET ( Figure 3C) and KDR in the BaF3 models correlate well with those generated using the respective phosphorylation endpoint assays thus maintaining the selectivity values previously observed ( Figure 3D). Accordingly, we introduced this as the routine POM screening assay with the option to further evaluate compound effects on phosphoprotein levels using the ELISA POM if necessary.

**Figure 4.**
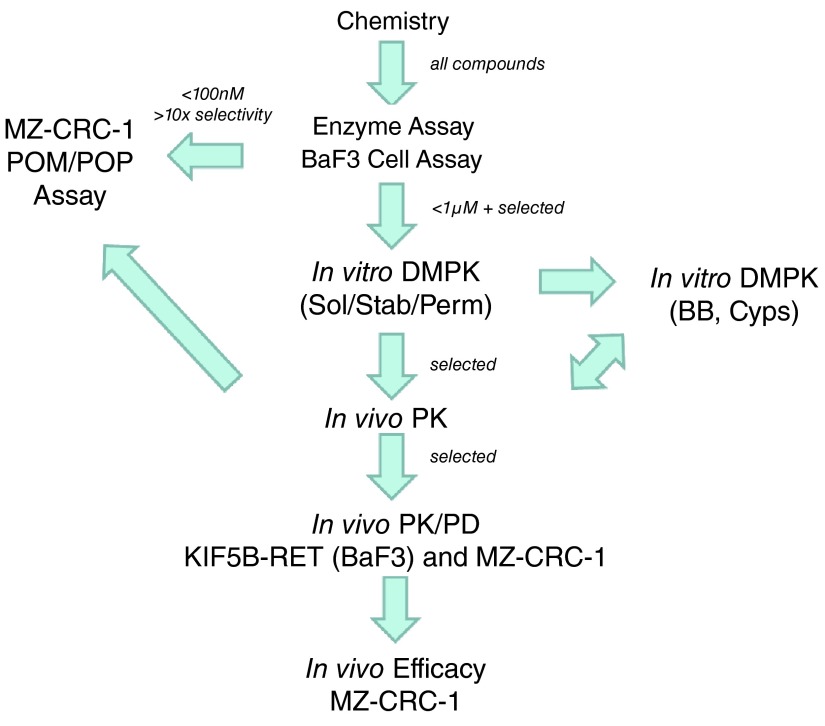
Current screening cascade (and selection criteria) for identification and development of potent and selective (vs KDR) RET inhibitors. Arrows represent compound flow. Sol = solubility, Stab = stability, Perm = permeability, BB = Blood-binding, Cyps = Cytochrome P450s.

**Supplementary Figure 3.**
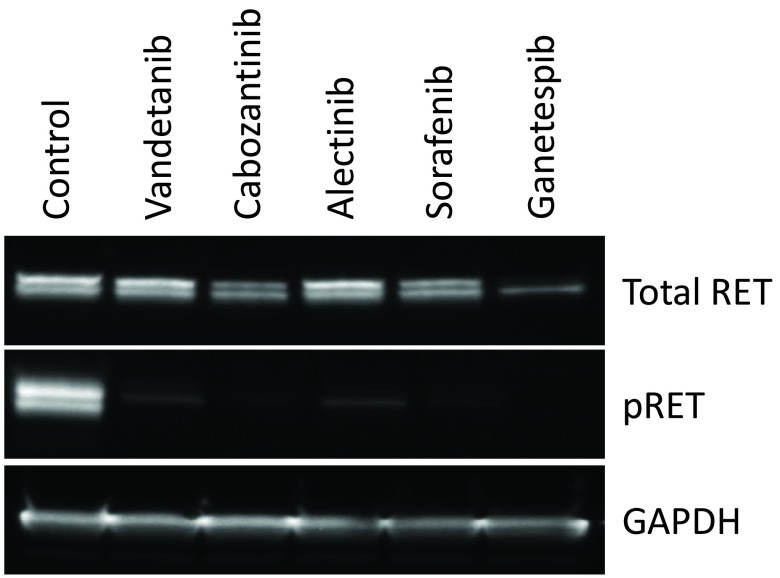
Degradation of RET protein following treatment with ganetespib. Western blot showing effects of compound treatment (1µM, 24 hours) on pRET, total RET and GAPDH levels in MZ-CRC-1 cells.

Chemical optimisation of the series ultimately led to the identification of a number of potent and selective RET inhibitors ( *e.g.* PDD16860 and PDD16964; Table 1). Clinical RET compounds currently under investigation in LAD were also profiled using the screening cascade ( Table 1). With the possible exception of alectinib, which it was not possible to dose at the highest concentrations due to solubility issues, none of the kinase inhibitors tested met our criteria for selectivity versus KDR ( *i.e*. >10x). Several reports in the literature ^21, 32^, which we have subsequently confirmed in-house (data not shown), demonstrate that alectinib is a much better cellular inhibitor of ALK (IC _50_ in H2228 cells = 10nM) than RET (IC _50_ in MZ-CRC-1 cells = 80nM). Given this, along with its limited solubility and required dosing of 300–600mg bid for clinical efficacy in ALK driven LAD, therapeutic inhibition of RET is unlikely at clinically relevant doses. Although not a direct inhibitor of RET activity, we have also profiled ganetespib, a HSP90 inhibitor. Ganetespib did reduce levels of active RET, through protein degradation ( Supplementary Figure 3) but was not RET-selective and exhibited more toxicity (toxicity margin = 0.7x) in the control non-RET driven HEK293 cells compared to the MTC RET-driven model, MZ-CRC-1. This indicates that the pleiotropic effects of HSP90 inhibition on numerous client proteins (including KDR) is likely to elicit non-specific toxicity at therapeutically active doses. Other aspects of the screening cascade ( Figure 4) are already in place and as discussed in an earlier publication ^1^, selected compounds have been assessed in *in vitro* DMPK assays as part of the chemical optimisation. Furthermore, *in vivo* models and capabilities ( Figure 4) have been established and validated (data not shown) and several tool compounds are currently undergoing assessment in PK/PD and efficacy studies. To date, our robust screening workflows have proved very successful in identifying potent, selective tool compounds and will continue to support our active pursuit of clinical candidates suitable for evaluation in RET fusion positive LAD patients.

**Table 1.** Clinical competitor and selected compound profiling data. The desired profile values indicate our target values for selectivity and toxicity margin. All values represent the geometric mean of at least four independent measurements. Figures are highlighted depending upon whether they easily (green); clearly do not (red) or almost (orange) meet the target criteria. PDD16860 and PDD16964 are examples of QZ compounds developed by screening through the cascade. ^1^Solubility of alectinib is limited, maximum dose achieved=1µM. ^2^Ganetespib is not a kinase inhibitor and therefore it is not possible to measure biochemical activity.

	Desired Profile	Vandetanib	Cabozantinib	Sorafenib	Sunitinib	Ponatinib	Lenvatinib	Alectinib	Ganetespib	PDD16860	PDD16964
**RET** **Enzyme** **IC _50_**		**0.05**	**0.65**	**0.13**	**0.03**	**0.023**	**0.024**	**0.18**	**^2^ND**	**0.0015**	**0.004**
**Selectivity** **vs. KDR**	**>100×**	**3.6×**	**0.02×**	**1.0×**	**0.03×**	**2.0×**	**0.05×**	**483×**	**^2^ND**	**250×**	**282x**
**RET Cell** **IC _50_**		**0.4**	**0.19**	**0.17**	**0.29**	**0.024**	**0.093**	**0.26**	**0.0039**	**0.12**	**0.26**
**Selectivity** **vs. KDR**	**>10×**	**1.6×**	**0.07×**	**0.11×**	**0.93×**	**0.2×**	**0.2×**	**^1^>3.8×**	**0.36×**	**23×**	**21x**
**Toxicity** **Margin**	**>>10×**	**35×**	**54×**	**13×**	**28×**	**127×**	**141×**	**^1^>35×**	**0.7×**	**19×**	**42x**

## Conclusions

We have established a robust screening cascade to enable the identification and development of a clinical candidate compound demonstrating potent and selective RET activity. Furthermore we have profiled clinical compounds currently under investigation for treatment of LAD confirming that they do not meet our target profile. We are continuing our efforts to develop a clinical candidate and will report on further progress in a future publication.

Datasets: selective inhibitors of RET and comparison with current clinical candidates through development and validation of a robust screening cascade This dataset includes all the raw data behind the figures shown in this paper. Information about each file can be found in 'Description of raw data files'. Click here for additional data file. Copyright: © 2016 Watson AJ et al. 2016 Data associated with the article are available under the terms of the Creative Commons Zero "No rights reserved" data waiver (CC0 1.0 Public domain dedication).

## Data availability

The data referenced by this article are under copyright with the following copyright statement: Copyright: © 2016 Watson AJ et al.

Data associated with the article are available under the terms of the Creative Commons Zero "No rights reserved" data waiver (CC0 1.0 Public domain dedication). 

*F1000Research*: Dataset 1. Datasets: selective inhibitors of RET and comparison with current clinical candidates through development and validation of a robust screening cascade, 10.5256/f1000research.8724.d122280 ^33^
