# Supplementary material for: Identification of selective inhibitors of RET and comparison with current clinical candidates through development and validation of a robust screening cascade
Source: F1000Res. 2016 Aug 23;5:1005. Originally published 2016 May 26. [Version 2] doi: 10.12688/f1000research.8724.2 (PMC4937820; doi:10.12688/f1000research.8724.2)
Supplement: Peer review history

# Peer review: Referee response for version 1

**DOI:** 10.5256/f1000research.9386.r14490

**Published:** 2016-07-11

**Authors:** Patrick A. Eyers

**Affiliations:** 1 Department of Biochemistry, Institute of Integrative Biology, University of Liverpool, Liverpool, UK

This well-controlled study from Watson and colleagues manages to avoid many of the cliches used in reporting kinase inhibitor studies ('potent and selective', 'highly specific'), and will be useful for those in the field interested in on and off-targets of clinical, probe and intermediate compounds, especially with relevance to RET, which is fast becoming an interesting drug target.

 The introduction is very clear, up-to-date with clinical and preclinical information, and leads into the new study efficiently.

 Minor points:

- One sentence mentioning or placing in context the M918T (P+1 loop) and V804M (gatekeeper) cancer-associated RET mutants would be useful for readers, since one is employed here.
- Is Km[ATP] the same for M918T RET, given that it autophosphorylates much more efficiently in vitro (Plaza-Menacho et al., Mol Cell 53:738?). Is Km literature, or measured in-house?
- EGFR = Epithelial Growth Factor Receptor, not endothelial.
- What is the evidence that RET inhibition by bandetanib and cabozantinib is actually 'secondary pharmacology' in terms of in vivo efficacy? This statement might be tempered.
- Cell POM: The POM assumes that the conversion of RET to pRET and KDR to pKDR in cells occurs inter/intramolecularly autophosphorylation (ie by the same protein kinase). Is this known, or surmised (reference would be useful).
- Please add a citation for the MZ-CRC-1 cell line
- Please add a citation, or Figure, demonstrating that HEK-293 cells do not express RET, since this is important for its use as control. What else is different between HEK-293 and MZ-CRC-1 though? Is this known?
- Table 1
  Challenging to distinguish between red and orange, how about another colour?
  Which cell line is this data from?
- Supplementary Figure 2.
  Although ganetespib (HSP90i) destabilising effects provides a clear positive control, cabozantinib or sorafentib (sub micromolar RET inhibitors) also destabilise RET (and abolish autophosphorylation). But is this also true for the most potent *in vitro* inhibitors Ponatinib, Lenvatinib, PD16860/16964, since definition of the IC _50_ value must require measurement of the measured RET total vs. pRET ratio? Elimination of RET, in addition to inhibition of activity might be a nice dual-approach here, and might influence selection of candidates?

I have read this submission. I believe that I have an appropriate level of expertise to confirm that it is of an acceptable scientific standard.

# Response

**Published:** 2016-07-15

**Authors:** Mandy Watson

**Affiliations:** Cancer Research-UK Manchester Institute, UK

Dear Patrick

 Thank you very much for reviewing this article. We appreciate your comments and suggested corrections and will address these in due course.

 Regards

 Mandy Watson

# Peer review: Referee response for version 1

**DOI:** 10.5256/f1000research.9386.r14492

**Published:** 2016-07-11

**Authors:** Kevin Hudson

**Affiliations:** 1 Oncology iMed, AstraZeneca Pharmaceuticals, Macclesfield, UK

I have read this article and I support indexation. Given my past experience in kinase drug discovery I believe I have an appropriate level of expertise to comment on the article. The article is scientifically sound and is written with sufficient detail and clarity to allow others to reproduce the work.

 The introduction section lays out a strong rationale for the efforts of this group to discover more selective RET inhibitors, indicating both the new emerging Oncology disease sector opportunities and the limited kinase selectivity of inhibitors currently in clinical use.

 The results and discussion section clearly describes the screening cascade used to discover more selective RET inhibitors. The correlations illustrated between the different assays helps build confidence in the screening cascade. In addition, the screening cascade  features different  mutant forms  (miss-sense, gene  fusions) of RET in addition to WT,  thereby indicating that compounds  the authors discover from this cascade have potential application across a  spectrum of tumours where RET  is an oncogenic driver. The data derived from testing of clinical agents in their cascade reinforces both the rationale behind the work and helps validate the cascade.    

 The direction the cascade provides for producing more selective RET inhibitors is largely focused around minimizing activity against the KDR receptor tyrosine kinase. This makes sense given the chemical start point for this work was vandetanib, a proven KDR inhibitor. However, as chemical series evolve during a drug discovery project, there is the risk of introducing additional secondary pharmacology (inhibition of additional kinases beyond RET and KDR). The author’s use of a ‘Toxicity Margin’ calculation based around cell proliferation sensitivity of RET-null HEK293 cells provides a rapid and simple measure to track  introduction of additional new pharmacology, although testing of frontrunner compounds in one of the widely available kinase panel screens may have been considered to fully appreciate the  kinase selectivity of the project compounds.   

 Minor comments / corrections:

- EGFR is an abbreviation for Epidermal Growth Factor Receptor, not endothelial growth factor as stated.
- VEGF treatment, not VEGFR treatment as stated
- The text relating to Figure 1B could more clearly indicate this data came from pre- incubation and wash out experiments.
- Figure 2C may benefit from more clearly labeling them as enzyme assays, i.e. RET enzyme (M918T) vs RET enzyme (WT).
- Other pertinent comments proposing clarification of statistical measurements used in the figures, and in wording in paragraph 3 of the Introduction, have already been noted by other reviewer, Anderson J Ryan.

 

I have read this submission. I believe that I have an appropriate level of expertise to confirm that it is of an acceptable scientific standard.

# Response

**Published:** 2016-07-15

**Authors:** Mandy Watson

**Affiliations:** Cancer Research-UK Manchester Institute, UK

Dear Kevin,

 Thank you very much for reviewing this article. We appreciate your comments and suggested corrections and will address these in due course.

 Regards

 Mandy Watson

# Peer review: Referee response for version 1

**DOI:** 10.5256/f1000research.9386.r14643

**Published:** 2016-07-07

**Authors:** Ian R. Hardcastle

**Affiliations:** 1 Newcastle Cancer Centre, Northern Institute for Cancer Research, Medical School, Newcastle University, Newcastle upon Tyne, UK

The article describes the development and validation of the screening cascade for selective RET kinase inhibitors used to identify selective inhibitors described in Ref 1. The rationale for pursuing selective RET inhibitors is clearly explained, and the clinical limitations of current pan-kinase inhibitors that inhibit RET laid out.

 The methods section has a well described rationale and is sufficiently detailed. The analysis of the data is sound and appropriate conclusions drawn. In particular, the method has accounted for the kinetics of binding, through time course experiments, and the possible impact of RET mutations in cell line studies. 

 The cascade developed adequately demonstrates selective cellular RET inhibition (POM). A good correlation is demonstrated between RET potency *in vitro* and growth inhibition in cell line models and this is taken as evidence of proof of mechanism.

I have read this submission. I believe that I have an appropriate level of expertise to confirm that it is of an acceptable scientific standard.

## References

[1] NewtonRBowlerKABurnsEMChapmanPJFairweatherEEFritzlSJGoldbergKMHamiltonNMHoltSVHopkinsGVJonesSDJordanAMLyonsAJNikki MarchHMcDonaldNQMaguireLAMouldDPPurkissAGSmallHFStowellAIThomsonGJWaddellIDWaszkowyczBWatsonAJOgilvieDJ: The discovery of 2-substituted phenol quinazolines as potent RET kinase inhibitors with improved KDR selectivity. *Eur J Med Chem*.2016;112: 10.1016/j.ejmech.2016.01.039 20-32 10.1016/j.ejmech.2016.01.039 26874741PMC4896931

# Response

**Published:** 2016-07-15

**Authors:** Mandy Watson

**Affiliations:** Cancer Research-UK Manchester Institute, UK

Dear Ian

 Thank you very much for reviewing this article.

 Regards

 Mandy Watson

# Peer review: Referee response for version 1

**DOI:** 10.5256/f1000research.9386.r14778

**Published:** 2016-07-05

**Authors:** Anderson J. Ryan

**Affiliations:** 1 Cancer Research UK/Medical Research Council Oxford Institute for Radiation Oncology, Department of Oncology, University of Oxford, Oxford, UK

This article presents a screening cascade for the identification of potent RET inhibitors with selectivity against VEGFR2 (KDR) that will be of general interest in the field. The background to the research is well described, and the methods sufficiently detailed to allow others to replicate the experimental procedures and the conclusions are balanced.  

Points

- EGFR is wrongly defined as ‘endothelial growth factor’
- ..toxicity …resulting from off-target kinases.  Should be on-target inhibition of non-RET kinases?
- … non-pharmacological toxicities….  Should be pharmacological toxicities associated with inhibition of non-RET targets?
- VEGF treatment does not affect levels of pRET
- Hek293 is more commonly HEK293
- ‘marvel’ is non-standard
- Figure 1.  Error bars not defined, n=??.  Should state this is RET enzyme assay
- Figures 2, 3, Suppl 2. Not stated what points represent [individual compounds??]
- Table 1. Confidence Interval values would be helpful as a surrogate of reproducibility, and support the claim of robustness, as reproducibility not specifically reported
- Table 1. Suggest including IC _50_ values for MZCRC1 cell proliferation above line for toxicity margin
- Figure 4.  Not clear why some arrows in figure are there.  Eg Arrow from *in vivo *PK to MZCRC1 POM/POP assay, or double-headed arrow from in vivo PK to in vitro DMPK
- Suppl Figure 1. No error bars (n=??)

I have read this submission. I believe that I have an appropriate level of expertise to confirm that it is of an acceptable scientific standard.

# Response

**Published:** 2016-07-15

**Authors:** Mandy Watson

**Affiliations:** Cancer Research-UK Manchester Institute, UK

Dear Anderson

 Thank you very much for reviewing this article. We appreciate your comments and suggested corrections and will address these in due course.

 Regards

 Mandy Watson
